# Chronic Medial Dislocation of the Proximal Ulna: A Rare Delayed Complication of a Supracondylar Fracture of the Humerus in Children

**DOI:** 10.7759/cureus.100371

**Published:** 2025-12-29

**Authors:** Said EL Orra, Raymond Massaad, Antonella Massaad, Dounia Massaad

**Affiliations:** 1 Orthopaedics and Traumatology, Beirut Arab University, Beirut, LBN; 2 Orthopedic Surgery, West Bekaa Hospital, Sohmor, LBN; 3 Orthopaedic Surgery, Carol Davila University of Medicine and Pharmacy, Bucharest, ROU

**Keywords:** avascular necrosis, case report, children, displaced supracondylar fracture, osteonecrosis, trochlea, ulnar dislocation

## Abstract

Osteonecrosis of the humeral trochlea is an uncommon but challenging complication of pediatric elbow fractures. We herein report a case of osteonecrosis of the trochlea, a rare and delayed complication of a supracondylar humerus fracture that led to a chronic dislocation of the ulna. A five-year-old boy arrived at the Emergency Room following a fall from a tree on an outstretched hand with an extended elbow. Radiographic studies of the right elbow confirmed the diagnosis of right supracondylar fracture. Open reduction and internal fixation by two Kirschner wires was carried out, followed by immobilization with a long arm cast for six weeks. Five years following surgery, the patient presented with cubitus varus deformity, with no pain or limitation of movement. Radiographic studies showed the presence of osteonecrosis of the humeral trochlea. At the 11-year follow-up consultation, the patient had no complaints, and physical examination of the right elbow showed a limited range of motion for flexion-extension. Radiographic studies showed a medial dislocation of the ulna. The patient was placed on long-term clinical and radiographic follow-up to monitor for secondary osteoarthritis. Our case emphasizes the importance of considering osteonecrosis of the trochlea and ulnar dislocation as delayed complications that can happen following supracondylar humerus fracture in the pediatric population. Clinical and radiologic follow-up are indispensable for diagnosing such a complication at an early stage.

## Introduction

Supracondylar humeral is considered the most common fracture in children below seven years and accounts for 15% of total pediatric fractures [1]. Up to two-thirds of hospitalizations in children are due to supracondylar fracture [2]. According to studies, it peaks at the age of six and is more common in males [1].

Complications following such a fracture include cubitus varus and valgus, fishtail deformity, and non-union [3]. An uncommon complication, not frequently mentioned in the literature, is the osteonecrosis of the trochlea [4]. The distal humerus is supplied by a single vessel that typically ends a few centimeters above the olecranon fossa [4]. Any disruption to this supply, whether due to a distal humerus fracture or following surgical intervention of such fractures, has been postulated as a contributing factor to tissue ischemia and eventual osteonecrosis [4]. Patients suffering from this late complication can present with complaints such as pain and decreased range of motion of the elbow [4]. Osteonecrosis is usually diagnosed through a radiographic plain imaging or a magnetic resonance imaging, and patients are managed conservatively or even surgically, if symptoms persist [4].

Although there are several case reports mentioning osteonecrosis as a late complication of such fractures, to our knowledge, chronic medial dislocation of the proximal ulna secondary to trochlear osteonecrosis has not been previously reported [5-8]. We report a case of a surgically treated supracondylar humeral fracture complicated by trochlear osteonecrosis. Chronic ulnohumeral dislocation subsequently developed and was believed to be a consequence of this complication.

## Case presentation

A five-year-old boy arrived at the Emergency Room of West Bekaa Hospital following a fall from a tree on an outstretched hand with an extended elbow. Patient complained of right elbow pain that was of sudden onset, aching in nature, aggravated by minimal elbow movement, and of 10/10 severity on a numeric rating scale.

**Figure 1:**
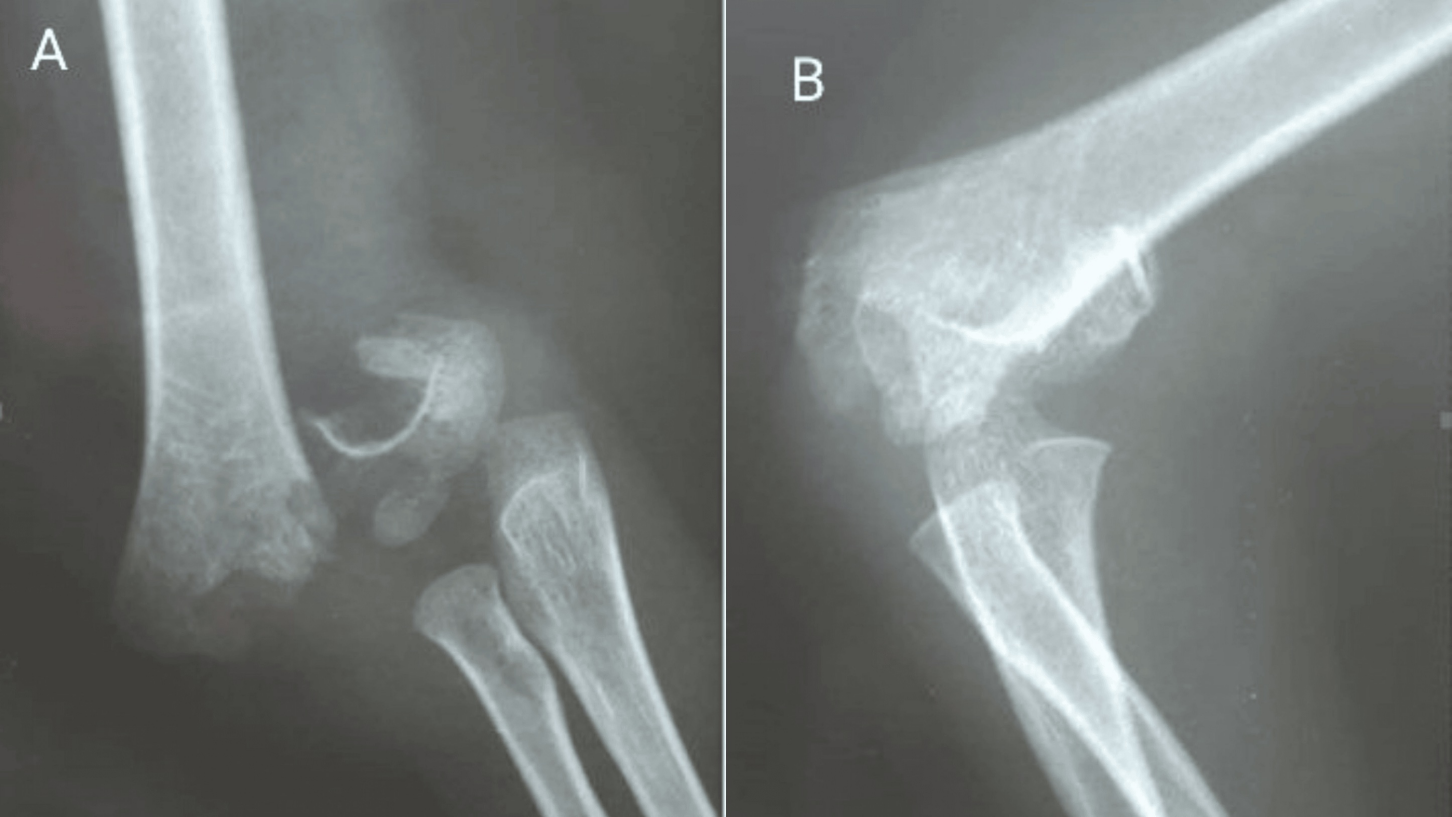
Right elbow X-rays, showing a type III supracondylar fracture A: Anteroposterior view. B: Lateral view

On physical examination, the patient was stable. The examination of the right upper limb showed scattered superficial abrasions. Swelling was noted at the cubital fossa, and a deformity was present at the level of the distal humerus. Tenderness was present at the right arm and elbow. The patient did not tolerate any elbow manipulation due to severe pain. Neurological examination of the radial, median, and ulnar nerves was normal. Brachial, radial, and ulnar pulses were present, with no evidence of ischemia. Radiographic studies of the right elbow showed a supracondylar fracture of the humerus (Gartland III) (Figures 1A, 1B).

**Figure 2:**
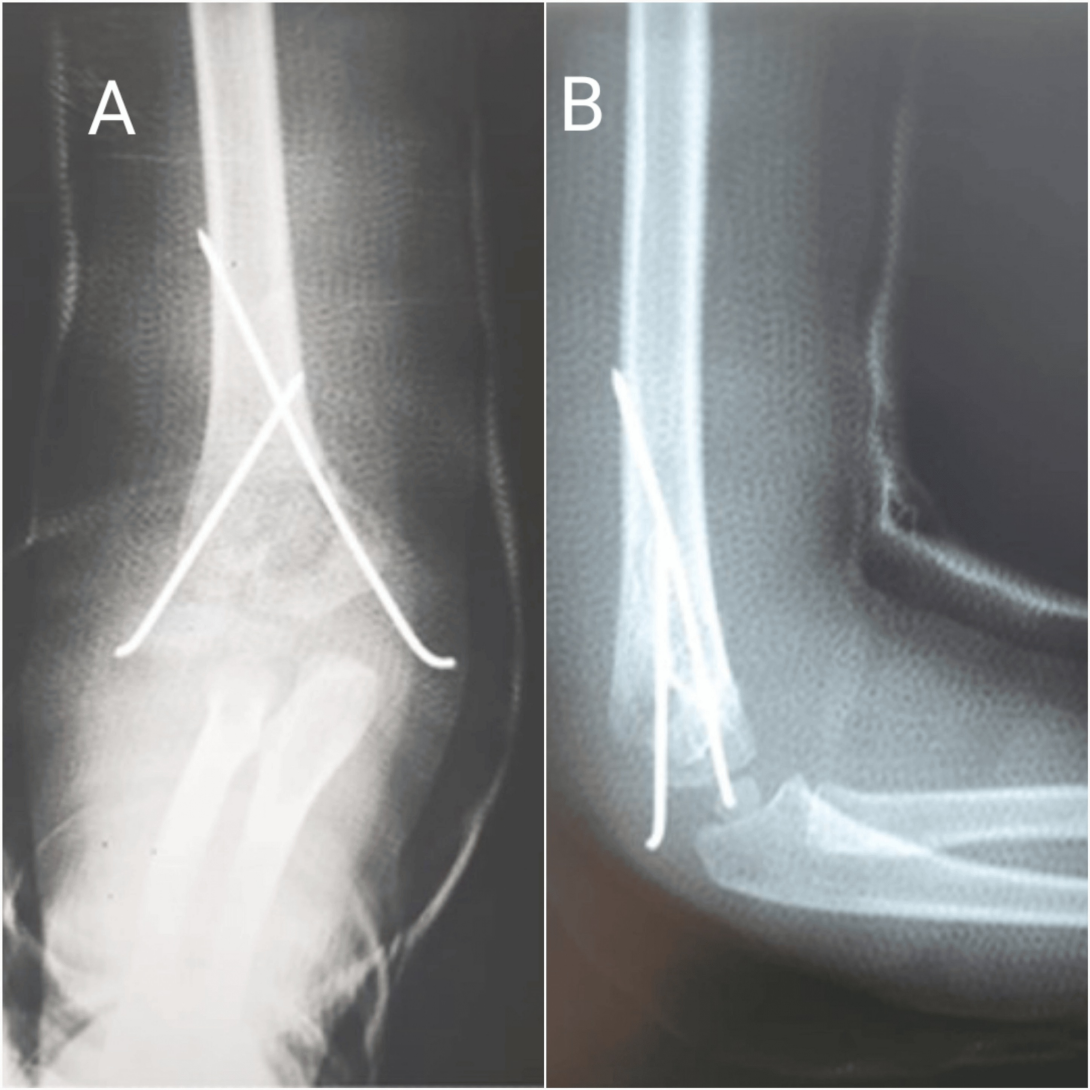
Post-operative X-rays of the right elbow demonstrating a satisfactory reduction following the fixation with two Kirschner wires A: Anterolateral view. B: Lateral view

Surgery was planned on the same day. After general anesthesia, and after an unsuccessful attempt to perform a closed reduction, we opted for an open reduction. After ensuring a satisfactory reduction on fluoroscopy imaging, fixation was achieved with two crossed Kirschner wires (Figures 2A, 2B).

**Figure 3:**
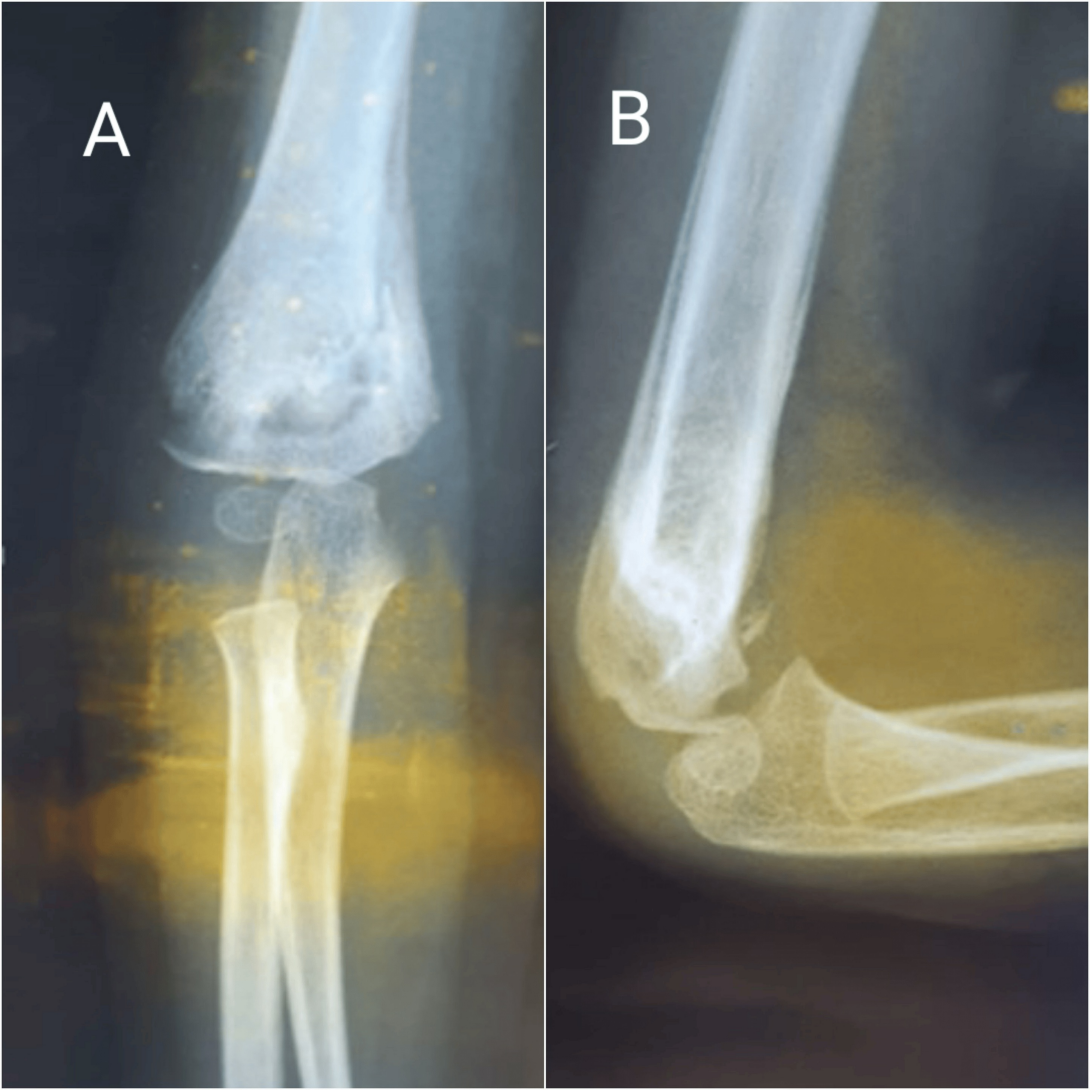
Right elbow X-rays at six-weeks follow-up, following the removal of the Kirschner wires A: Anteroposterior view. B: Lateral view

The surgery was uneventful, and a long arm cast was applied. We opted for the removal of the Kirschner wires at six-week follow-up, after ensuring an adequate consolidation on X-rays (Figures 3A, 3B).

**Figure 4:**
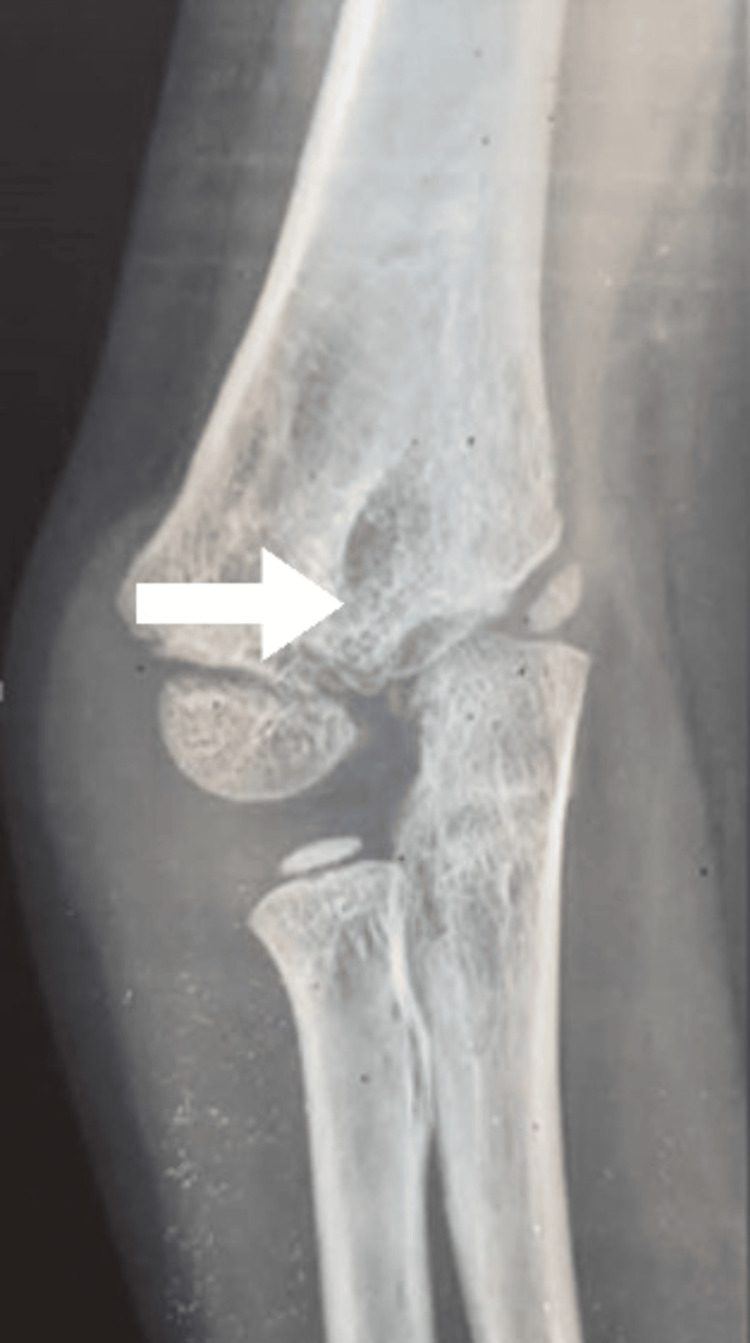
Anteroposterior X-ray of the right elbow at five-year follow-up Note the lucency involving the trochlea suggesting osteonecrosis (arrow)

A follow-up was done after six weeks, showing a full range of motion of the elbow. Nerve examination was normal. Five years after surgery, the patient presented to our clinic complaining of a deformity of his elbow, but denied any pain during this period. On examination, a cubitus varus deformity was noted, but with no limitation in the range of motion of the elbow. X-rays of the right elbow showed lucency involving the trochlea, suggesting osteonecrosis of the trochlea (Figure 4). We decided to maintain a conservative treatment with periodic follow-ups.

**Figure 5:**
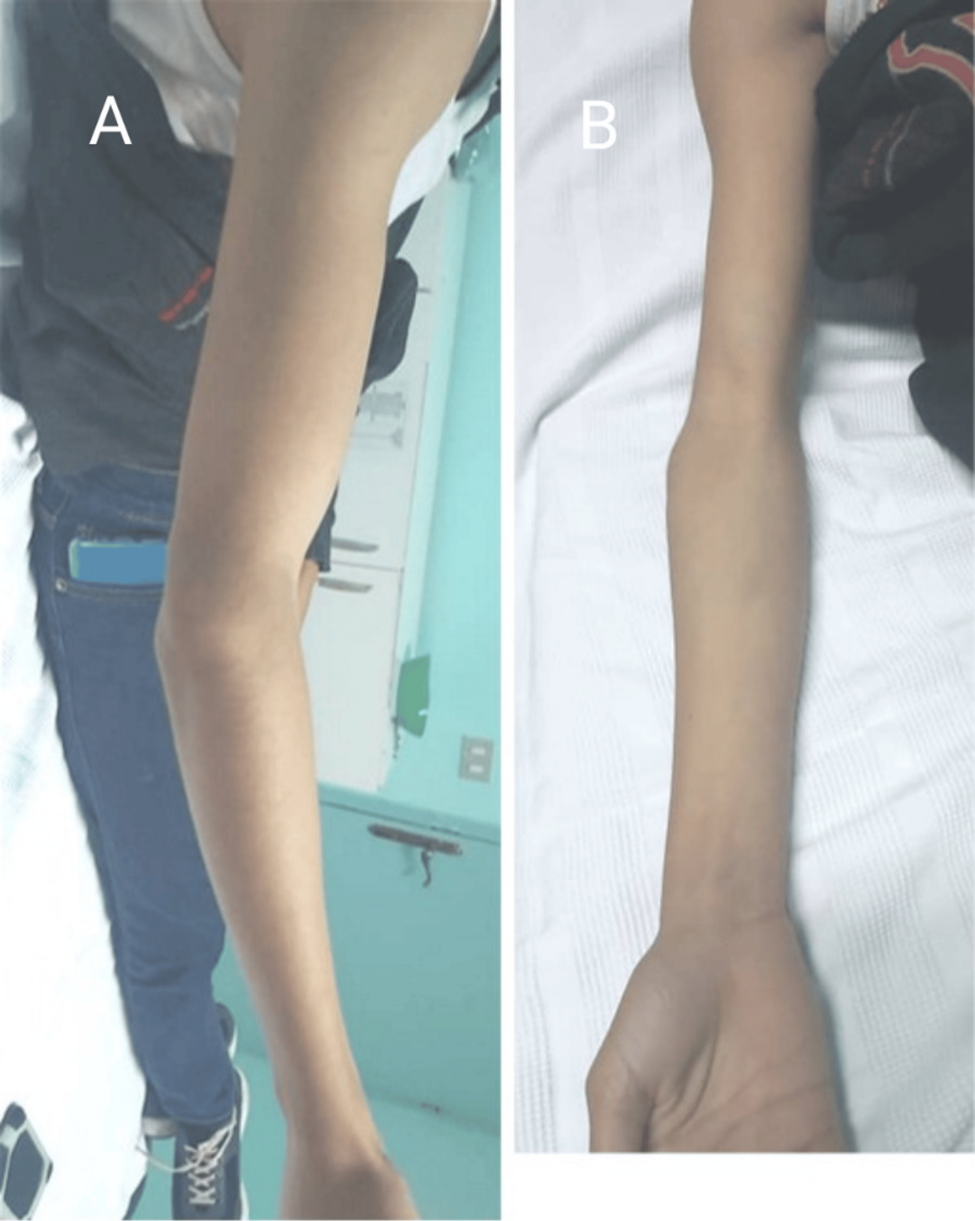
Picture of the right arm at 11-year follow-up Note the limitation to achieve full elbow extension. A: Lateral view. B: Anterior view

**Figure 6:**
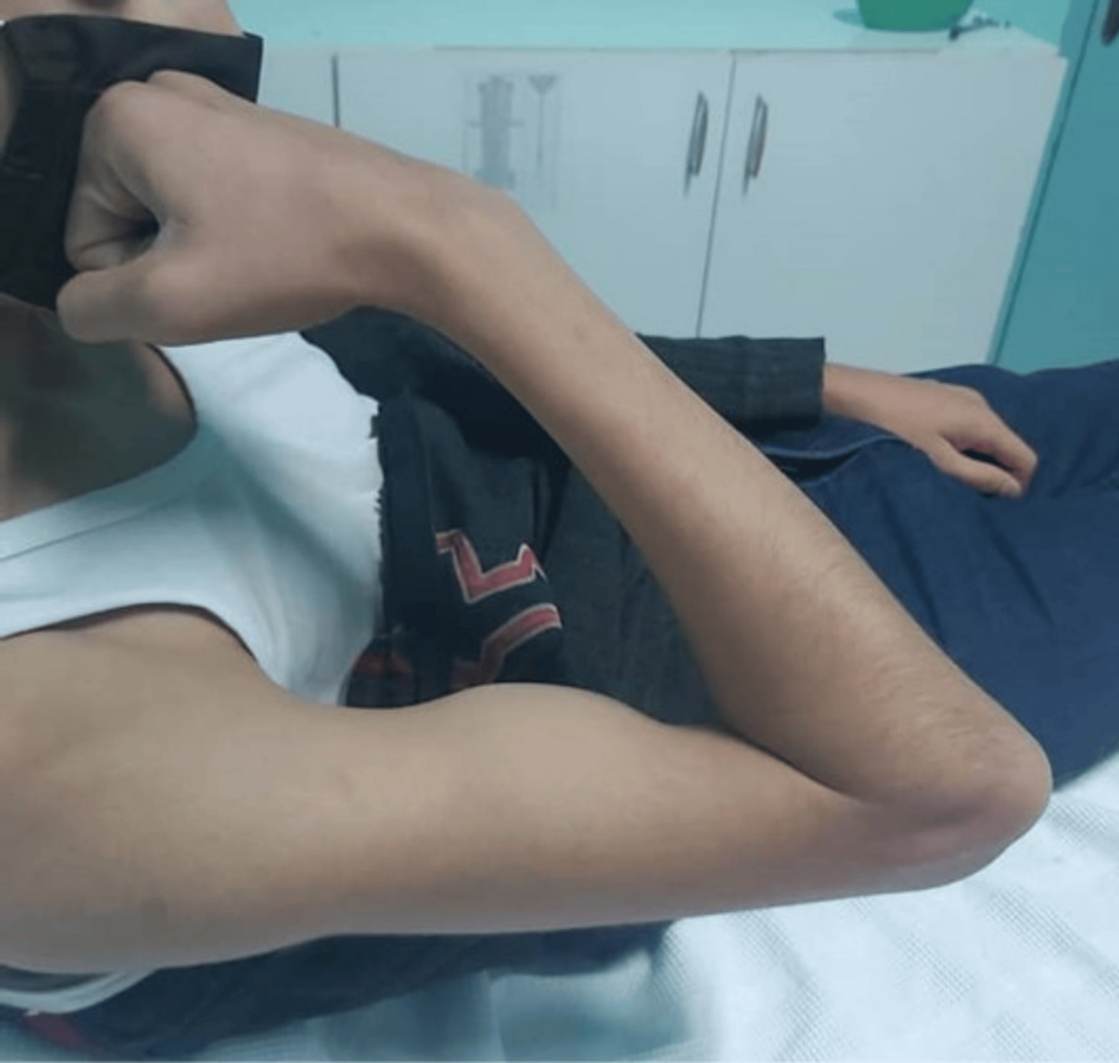
Picture of the right arm at the 11-year follow-up Note the inability to achieve full elbow flexion

**Figure 7:**
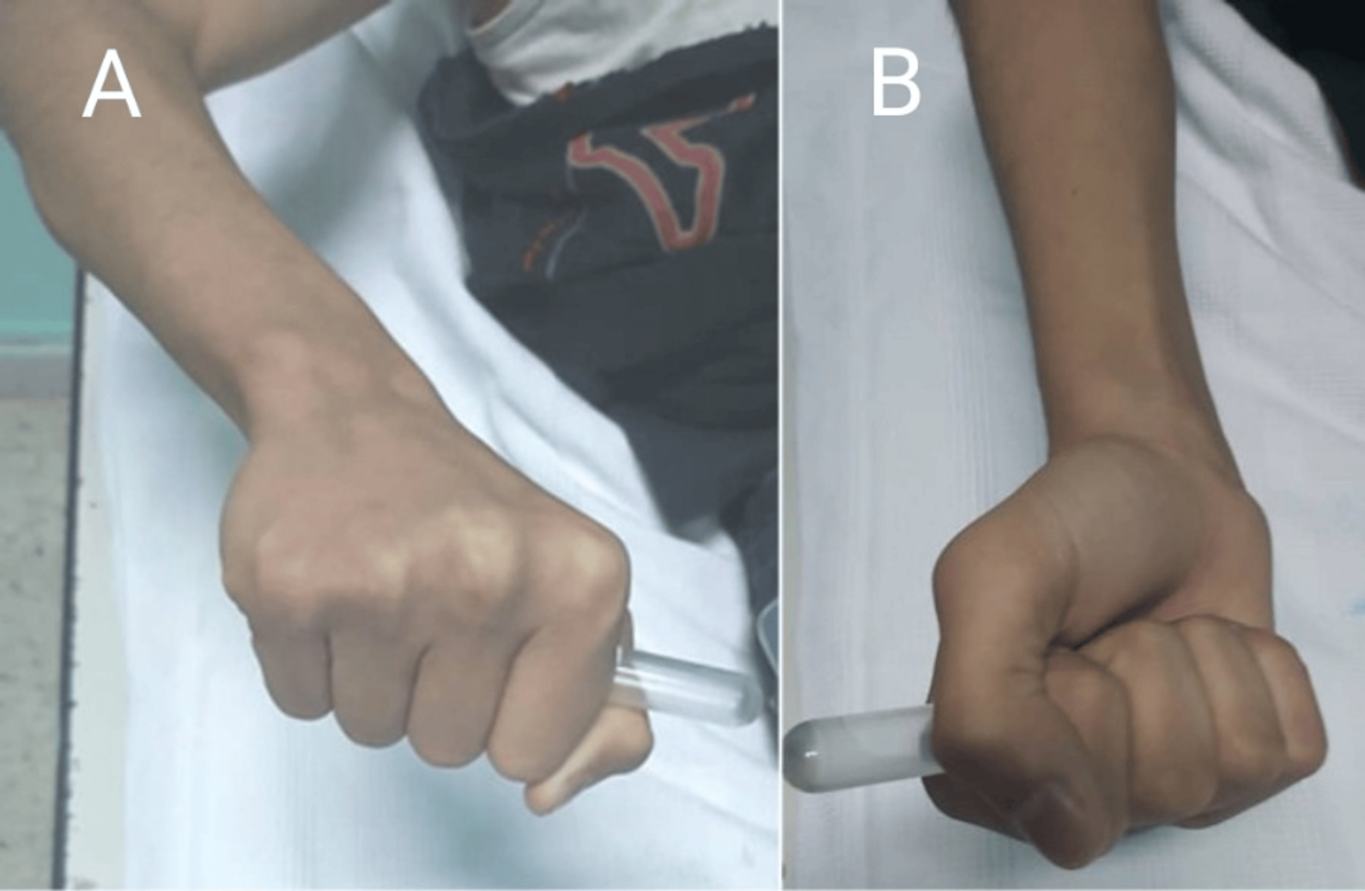
Picture of the right forearm at 11-year follow-up, demonstrating adequate range of motion in pronation and supination A: Forearm fully pronated. B: Forearm fully supinated

Eleven years after surgery, the patient, aged 16 years, returned for follow-up with no new complaints. Upon examination of the right elbow, we noticed a limited range of motion of the elbow (25°-130°) (Figures 5A-5B, Figure 6), but with full ability of pronation and supination (Figures 7A, 7B).

**Figure 8:**
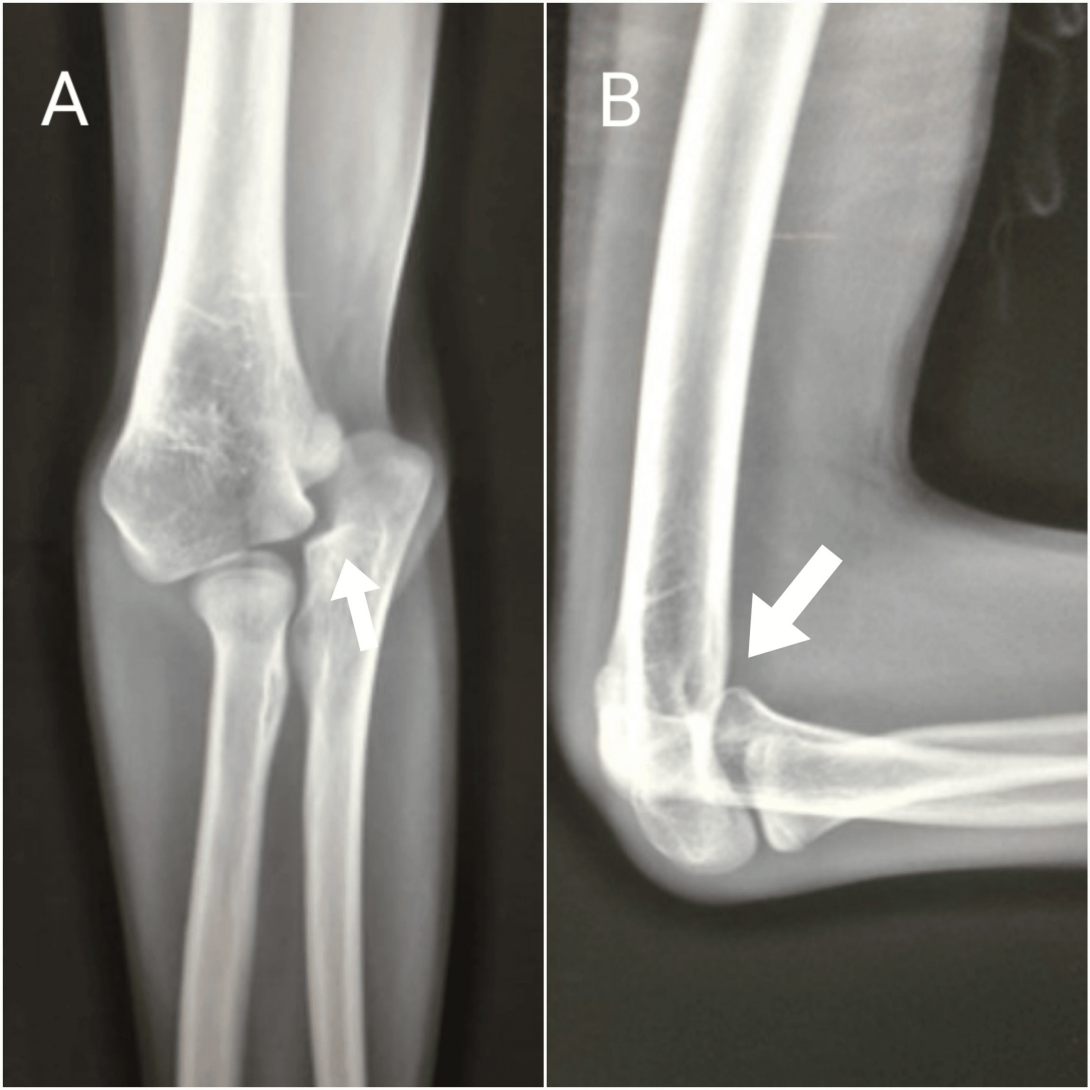
Right elbow X-rays at the 11-year follow-up, showing a preserved radio-humeral allignment Note the medial dislocation of the proximal ulna (arrow). A: Anteroposterior view. B: Lateral view

X-rays of the right elbow showed a medial dislocation of the proximal ulna with preserved radio-humeral alignment (Figures 8A, 8B). Considering that the patient had no complaints regarding his range of motion and had adequate function of his limb, we opted to maintain a conservative approach. The patient was put on a long-term follow-up schedule for the detection of early signs of osteoarthritis.

## Discussion

Supracondylar fracture of the humerus was classified by Gartland as type I (non-displaced), type II (partially displaced), and type III (completely displaced) [4]. According to the mechanism of injury, supracondylar fractures are classified into extension or flexion injuries. The extension type (90-98% of cases) is caused by a fall on an outstretched hand with the elbow extended. The distal fragment in this type could be displaced posteromedially (90% of cases) or posterolateral (10% of cases) [2]. In our case, the fracture was of Gartland type III, extension injury.

Treatment of supracondylar fractures depends on Gartland classification: type I are managed by immobilization; type II are managed by either closed reduction and immobilization, or by fixation using percutaneous Kirschner wires; and type III is usually managed by closed reduction and percutaneous fixation by Kirschner wires [9]. Open reduction and fixation by Kirschner wires is indicated in cases of open fractures, vascular injury, or irreducibility [10]. In our case, we opted for the open reduction and fixation due to the irreducibility of the fracture.

Complications following supracondylar fractures vary from one patient to another. A study of 291 children showed that only 4% had long-term deformities, where most of these deformities were cubitus varus (3%), and the minority were cubitus valgus (1%). Corrective surgery was needed in 1% [10]. In another study done in 200 children with type III extension injuries, 9.5% were associated with neurovascular injury, with the anterior interosseous nerve as the most common nerve injured [11]. However, according to Abbott et al., no correlation has been found between the complications and the timing of surgery [12]. One of the complications, not commonly mentioned in the literature, is the osteonecrosis of the trochlea.

Osteonecrosis of the trochlea was first mentioned by McDonnell and Wilson in 1948; osteonecrosis was diagnosed in four out of 53 patients who had a supracondylar fracture, with the patients having clinical presentation after two to seven years following the injury. Osteonecrosis is believed to be associated with two factors: multiple reduction attempts and narrowing of the joint space with thinning of the cartilage. The etiology still remains unclear; however, vascular and idiopathic growth disturbances have been proposed as possible etiologies [13]. The vascular supply to the elbow is rich; however, the lateral part of the trochlea and capitellum are considered as watershed areas, as both rely on end arteries penetrating the posterior part of the humerus. Hence, an iatrogenic injury to the vascular supply can lead to osteonecrosis [14].

A mean interval of 33 months between the fracture and onset aligns with findings typical of osteonecrosis. Patients with osteonecrosis of the trochlea present with pain, restricted range of motion, or crepitus. Any suspicion of osteonecrosis should be investigated by radiographic images or magnetic resonance imaging. It is important to note that the medial trochlea in children younger than seven to eight years is still cartilaginous, and therefore osteonecrosis will not be evident on radiographic imaging in this group of population [4].

In our patient, the dislocation of the ulna presented a rare complication after a supracondylar fracture in children, due to the complete necrosis of the lateral trochlea, which led to the loss of the congruity of the ulnohumeral joint and subsequent dislocation. The initial displacement of the fracture and the open reduction may have contributed to the development of osteonecrosis.

Conservative procedure can be attempted depending on the severity of the presenting symptoms. Keeping the patient under observation is important. Surgical management, represented by debridement, either arthroscopic or open, combined with capsulotomy, osteotomy, and epiphysiodesis, can be attempted in patients with persistent symptoms [4].

Fortunately, our patient was free of pain and had no complaints despite a 25° extension lag and 10-15° flexion deficit. A long-term follow-up was necessary to detect any signs of early osteoarthritis.

## Conclusions

Supracondylar humerus fractures occur predominantly in pediatric elbow injuries. However, although rare, delayed complications can happen, such as the osteonecrosis of the trochlea and subsequent ulnar dislocation, which can lead to a limited range of motion of the elbow. Although, in our case, we opted for conservative management as the patient did not have a major functional impact, a close clinical and radiologic follow-up are indispensible to diagnose such a complication at an early stage.
